# Transmission Electron Microscopy as a Powerful Tool to Investigate the Interaction of Nanoparticles with Subcellular Structures

**DOI:** 10.3390/ijms222312789

**Published:** 2021-11-26

**Authors:** Manuela Malatesta

**Affiliations:** Department of Neurosciences, Biomedicine and Movement Sciences, University of Verona, I-37134 Verona, Italy; manuela.malatesta@univr.it

**Keywords:** nanomedicine, nanoparticle uptake, nanoparticle biodistribution, ultrastructure, correlative microscopy, histochemistry, microanalysis

## Abstract

Nanomedical research necessarily involves the study of the interactions between nanoparticulates and the biological environment. Transmission electron microscopy has proven to be a powerful tool in providing information about nanoparticle uptake, biodistribution and relationships with cell and tissue components, thanks to its high resolution. This article aims to overview the transmission electron microscopy techniques used to explore the impact of nanoconstructs on biological systems, highlighting the functional value of ultrastructural morphology, histochemistry and microanalysis as well as their fundamental contribution to the advancement of nanomedicine.

## 1. Introduction

Since nanotechnological research turned its attention to the biomedical field and nanomedicine was born [1], the development of new nanoproducts has necessarily involved the study of their interactions with the biological environment. Whatever the nanoconstructs are intended for, e.g., as drug delivery systems, contrast agents, biosensors, sorting systems or scaffold components [2,3,4,5,6,7,8,9], the knowledge of their structural and functional interactions with cells, tissues and organs is essential to ensure both safety and efficacy. Imaging techniques have played a primary role in meeting this need.

In vivo imaging techniques (e.g., magnetic resonance imaging, optical imaging, positron emission tomography) have been widely applied, frequently in combined approaches, to verify the targeting, biodistribution and clearance of the nanoconstructs in the whole organism [10,11,12,13,14]. They are particularly suitable for the dynamic tri-dimensional tracking of nanoparticulates and for longitudinal studies but they are characterized by a low resolution (in the order of hundreds of micrometers), which prevents the study of the spatial and functional relationships at a histological and cytological level. Light and electron microscopy have mostly been used to monitor the uptake and relocation of nanoparticles inside tissues and cells [15,16,17,18,19,20]. 

Fluorescence microscopy (both conventional and confocal) is the most popular approach in nanomedical research due to the relatively simple experimental protocols and the possibility of investigating wide cell populations or relatively large tissue samples to obtain 3D information and to perform dynamic live observations. However, its resolution (approximately 200 nm) is insufficient to detect single nanoparticles and observe their interactions with the structural components of tissues or cells; moreover, both nanoconstructs and cytological/histological components need to be fluorescently labelled to become visible so that the detection of nanoparticulates relies on marker binding that can fail in the biological environment due to molecular interactions (especially enzyme activity). Super-resolution microscopy has significantly improved the capabilities of fluorescence imaging but its application in nanomedical research is still limited due to the experimental complexity and high costs [21,22,23,24]. In addition, super-resolution techniques do not overcome the limitations related to indirect fluorophore-mediated detection.

Scanning electron microscopy (SEM) utilizes a focused beam of electrons that scan the surface of the sample, providing images with a three-dimensional appearance. Despite the high resolution (3–20 nm), imaging is restricted to the surface of the sample. For this reason, SEM has mostly been used in nanomedical research to characterize the spatial relationships between nanoparticulates and the cell surface with particular reference to the internalization process and cell shape modification [25,26,27,28] although field emission SEM (using a high energy beam of electrons) has allowed the visualization of nanoparticles also in the endosomal compartment [29].

Transmission electron microscopy (TEM) provides images obtained by a beam of electrons transmitted through a thin specimen, thus allowing the detailed visualization of the interior of the sample. This microscopy technique has widely been used in nanomedical research and is able to reveal the fine relationships between nanoparticulates and cell/tissue components due to the unique information provided by its high resolution. Thanks to the very short wavelength of the electron beam (100,000-fold shorter than the photons in the visible spectrum), a sub-nanometer resolution can be achieved corresponding with approximately 0.2 nm in conventional TEM. However, biological samples need to be appropriately processed to be observed with a transmission electron microscope and this preparation may limit the resolution to approximately 2 nm [30]. For example, resin embedding causes a noise, which become larger with the increasing section thickness whereas cryofixed and cryosectioned samples, where resin embedding is omitted, must be protected by a methylcellulose layer (Tokuyasu technique) that may decrease the image quality. Therefore, to observe biological samples in TEM, it is necessary to set up preparation procedures suitable to match the structural and/or molecular preservation with the resolution. Despite the sample processing limitations, the TEM resolution remains significantly higher in comparison with light microscopy; moreover, it may often allow the direct visualization of nanoconstructs and cell/tissue components without recourse to markers [31,32,33,34]. The TEM techniques also have their drawbacks; the microscope and the related equipment are more expensive than those required for light microscopy, the sample processing is time consuming and must be performed by skilled personnel and observations can only be made on small and very thin (usually 70–90 nm) sample slices. In addition, only “static” information can be obtained due to the physical/chemical fixation and resin embedding of the sample, which precludes dynamic studies. Despite these caveats, TEM remains the technique of choice to finely study the interactions of nanoconstructs with the biological environment.

The present review article aims to overview the TEM techniques used to explore the impact of nanoconstructs on biological systems, highlighting their fundamental contribution to the advancement of nanomedical research. Attention will not be paid to the extensive use of TEM for studying the structural features of nanoconstructs in the course of their manufacture or functionalization.

## 2. The Functional Value of Ultrastructural Morphology in Nanomedical Investigations

During the process of developing and validating novel nanoconstructs for biomedical use, the safety assessment is chiefly important and cannot be limited to the simple evaluation of the cell death rate following administration. The sub-lethal cell stress or the organelle damage must also be considered because the cell injury may alter the tissue homeostasis and trigger inflammatory responses in the organisms receiving the nanomedical product [35,36,37].

In this view, TEM can proficiently be used; alterations of the plasmalemma (such as blebbing or loss of surface protrusions), the reorganization of the membranous endoplasmic systems, the shrinkage or swelling of mitochondria (with changes or loss of the cristae), the accumulation of residual bodies or the rearrangement of the nuclear domains are all hallmarks of different degrees of cell stress that are barely detectable in light microscopy [38,39,40,41,42,43,44]. Fine histological changes such as the thickening or disruption of the basement membranes, the alteration of the capillary walls or the restructuring of the extracellular matrix may also reveal tissue stress or damage after nanoparticle administration [45,46,47,48,49]. 

However, the most relevant contribution of TEM to the development of new nanomedical devices is the capability of elucidating their interactions with tissues and cells. The localization and detailed tracking of nanoparticulates in the biological environment from their uptake to their intracellular degradation is basic information to design efficient administration strategies for therapeutic and diagnostic purposes. 

When nanoconstructs are tested ex vivo or in vivo, a fine analysis of their biodistribution is essential to predict their biomedical potential. The knowledge of the tissue components where they localize (i.e., specific cell types, the amorphous or fibrillar components of the extracellular matrix and the blood vessels) and the biological barriers they are able to cross allows the selection of the most suitable carriers as well as the appropriate administration routes and protocols. In the literature, a large number of articles have been published where TEM was used to detect the nanoparticulates inside tissues and organs [45,50,51,52,53,54,55,56,57,58]. 

At the cellular level, TEM can provide unequivocal information on the mechanism(s) allowing nanoparticles to enter the cells by crossing the plasma membrane, provided that the sample had been processed appropriately to preserve their spatial relationship with the cell surface [59,60]. This knowledge is extremely useful for the design of efficient delivery systems. TEM has demonstrated that endocytosis is the most common uptake mode; nanocarriers have been observed making contact with the plasma membrane occurring in plasma membrane invaginations and entering the cell enclosed in endosomes [59,61,62,63,64,65,66,67,68,69,70]. Endocytosis may take place for single nanoparticles and also for small nanoparticle groups whereas single large nanoconstructs or large clusters of nanoparticulates enter the cells by means of phagocytosis or macropinocytosis, respectively [63,71,72,73,74]. All these uptake mechanisms may be facilitated by several types of receptors [20,75,76,77]. Ultrastructural observations showed that a few nanoconstructs may cross the biological membranes in the absence of endosomal structures [46,72,78]. This phenomenon has mainly been observed for lipid-based or lipid-coated nanocarriers, which likely fuse with the plasma membrane [79,80,81,82] although conclusive ultrastructural evidence of this occurrence has not been given so far. 

The uptake mechanism affects the intracellular fate of the internalized nanoconstructs as it can clearly be visualized by TEM. Endosomes/phagosomes are destined to fuse with primary lysosomes with the consequent degradation of the contained nanoparticles by the action of lytic enzymes [83,84]. Therefore, the nanoconstructs internalized via endocytosis/phagocytosis will generally be trapped in vacuolar structures without making contact with any organelle; only the degradation products that can cross the lysosomal membrane will spread to the cytosol whereas nanoparticle remnants will remain inside secondary lysosomes and residual bodies [61,62,63,64,65,66,67,68,72,73,85,86,87].

TEM images have also showed that several nanoconstructs are able to escape the endosomes and then occur freely in the cytosol, avoiding lysosomal degradation. This was first observed for cationic nanoconstructs and was explained by the so-called “proton sponge effect” [88,89,90] but recently, anionic nanocomplexes [91] and neutrally charged nanoparticles [92] have also been proven to efficiently escape endosomes. Endosomal escape has also been observed for gold nanoparticles coated with Listeriolysin O toxin, which is able to perforate the endosomal/lysosomal membrane [93] and, in prickly nanodiamonds, is able to break the endosome membrane [94]. On the contrary, lipid-based nanoparticles undergo endosomal escape via membrane fusion without the disruption of the membrane [95,96]. The occurrence of endosomal escape is essential information to understand the intracellular dynamics of the nanoparticulate as well as the availability of the eventually loaded molecules; the occurring cell damage following endosome breaking must carefully be considered to prevent undesirable side effects. Presently, TEM is the only tool able to provide direct evidence for these phenomena.

Once free in the cytosol, the nanoconstructs (either escaped from the endosomes or internalized by routes other than the endocytic one) may persist therein for variable periods of times and directly interact with cell organelles before undergoing degradation processes depending on their chemical nature [97,98]. 

As an example, phosphatidylcholine-based nanoparticles were found to be rapidly degraded by cytosolic enzymes of different cell types [99,100], thus providing an explanation for the concomitant accumulation of lipid droplets as storage sites for the nanoparticle hydrolytic products. TEM, therefore, definitely clarified the reason for the high biodegradability of these organic nanoparticles and paved the way for studies on their therapeutic use. Similarly, liposomes and solid lipid nanoparticles were observed to rapidly disassemble in the cytosol where their degradation products were found to accumulate inside lipid droplets, probably due to a chemical affinity [46,72,96,101]. Again, TEM provided a demonstration of the physiological and safe degradation of these nanoconstructs, allowing also the explanation of the rapid disappearance of these nanoconstructs observed in light microscopy. 

Several nanoparticulates free in the cytosol may also enter the cell nucleus [87,98,102,103,104,105], probably by passing through the nuclear pores [106] or by being entrapped in the reassembling nuclear envelope at the end of mitosis [87,107]. The presence of nanoparticles inside the nucleus is potentially harmful because the interaction of the nanomaterials with the nucleic acids and/or the nuclear factors may have unpredictable consequences on gene expression and RNA processing. This information is, therefore, crucial to evaluate the biological impact of a nanoconstruct and TEM is the most suitable tool for obtaining unequivocal evidence of the intranuclear distribution and entry mechanisms. 

TEM is also essential to understand the fate of the nanoconstructs that occur freely in the cytosol but do not undergo a complete degradation, thus providing information on their intracellular persistence. Endosomal-escaped nanoparticles were observed to undergo exocytosis [108,109,110] but they can also re-enter the lytic pathway by the autophagic route [72,87,96,101,111]. TEM has allowed the observation of non-degradable nanoparticle remnants persisting inside vacuolar structures [61,62,64,65,66,67,68,72,73,86,87,112], thus providing unique information on their biodegradability. It is worth noting that fluorescent microscopy does not allow entire nanoparticles to be distinguished from their remnants, provided that they keep their binding with the marker. The knowledge of the intracellular persistence of nanocarriers is crucial to plan administration strategies that avoid the risk of adverse effects, especially when multiple administrations are needed.

## 3. Combined Electron Microscopy Techniques for Nanoparticle Tracking

The combination of conventional TEM with other imaging or analytical techniques has allowed a deepening of the knowledge of the relationships between nanoconstructs and the biological environment.

Correlative light and electron microscopy (CLEM) has offered important benefits to nanomedical research due to its capability of matching the advantages of light microscopy in giving a general and dynamic view of the cell with the ultrastructural detailed information provided by TEM [113,114,115,116,117]. Fluorescence microscopy in combination with TEM and an ion beam analysis (IBA, which allows the evaluation of the chemical elemental distribution) has allowed the detection, tracking and quantification in vivo and at a high resolution of TiO_2_ nanoparticles inside human keratinocytes, thus revealing the mechanisms responsible for their cytotoxicity [118]. The combination of live cell imaging and TEM revealed the intracellular distribution dynamics and the fine relationships with cell organelles of silver nanoparticles as well as the absence of ultrastructural alterations due to their administration in the frame of a study aimed at identifying novel candidates for an antiviral therapy [105]. Combining fluorescence microscopy, live cell imaging, magnetic force microscopy and TEM, the intracellular behavior of Fe_7_C_3_@C superparamagnetic nanoparticles was elucidated and their suitability as a novel contrast agent was evaluated [119]. 

One of the technical limitations of conventional TEM is the possibility of analyzing only thin-sectioned samples, thus obtaining two-dimensional images only. Thanks to tomographic approaches, this limitation has been overcome [120]. Transmission electron tomography is based on the 3D reconstruction of several images acquired from a single sample section at incremental angles with an axial resolution of approximately 2–8 nm. The analyzable volume is restricted by the section thickness and the scattering of the transmitted electrons decreases the image quality in samples thicker than 300 nm. However, a 3D reconstruction may be implemented by combining serial sections (serial tomography) although this is complicated and time consuming. The tomography of thicker samples (up to 1.5 µm) may be obtained using scanning transmission electron microscopy (STEM) with a spatial resolution of 5–10 nm [121,122]. Detailed ultrastructural 3D information is achievable also by using high resolution field emission SEM on resin embedded samples; focus ion beam scanning electron microscopy (FIBSEM) uses a focused ion beam to mill the sample surface and obtain serial images with a z-axis resolution of 3–30 nm whereas serial block face scanning electron microscopy (SBFSEM) uses a built-in diamond knife to cut serial sections that are then imaged with a z-axis resolution of 20–50 nm. Both techniques allow the fine analysis of larger volume samples than TEM and STEM with a good z-axis resolution (especially FIBSEM) but are characterized by a lower x- and y-axis resolution (3–30 nm) that entail the destruction of the observed sample [120].

With the aim of understanding the detailed 3D spatial relationships of nanoparticles with cell and tissue components and obtaining quantitative data on their 3D distribution, several high technological approaches based on these EM tomographic techniques have been applied to nanomedical research. TEM and 3D electron tomography were used to track the intracellular pathways of superparamagnetic iron oxide nanoparticles [123]. Conventional TEM and STEM were combined to visualize the 3D distribution of different gold nanoparticles in whole cells [124,125]. FIBSEM sectioning was used to correlate 3D surface cell imaging with the 2D TEM intracellular visualization of nanoparticles [126,127]. The combination of TEM and SBFSEM allowed the quantification of the intracellular uptake of quantum dots [128]. EM tomographic techniques have also been used in association with CLEM. Fluorescence microscopy and STEM tomography allowed the analysis of 900 nm-thick sample sections, thus providing the 3D distribution of rhodamine B-labelled gold nanoparticles in human retinal pigment epithelial cells [129]. Similarly, quantum dots were identified inside fibroblasts [130] and human pathological tissues [131]. STEM in combination with light microscopy allowed the visualization of molybdenum-based nanoparticles into human hepatoma cells cultured in 2D (cell monolayers) and 3D (spheroids) models [132]. Cryo-soft X-ray tomography and TEM provided information on the 3D interactions of superparamagnetic iron oxide nanoparticles with breast cancer cells [133]. The combination of dynamic confocal imaging, low resolution TEM and dark-field STEM allowed the description of the uptake and intracellular distribution of ZnO-based nanoparticles [134]. Correlative microscopy methods comprising optical microscopy, TEM, SEM, helium ion microscopy (HIM) and FIBSTEM were used to determine the number and 3D distribution of gold nanoparticles in a whole cell volume [135]. Fluorescence microscopy was combined with scanning transmission X-ray tomography and ptychography to obtain the 3D distribution in the intracellular environment of nanoparticles made of an Fe_3_O_4_ core coated by a fluorescent SiO_2_ shell [136].

A further advantage of TEM in nanomedical research is the possibility of associating fine structural imaging with an in situ chemical analysis. This not only allows nanoparticles to be unequivocally detected inside cells and tissues but also provides qualitative and quantitative data on their chemical composition as additional location markers and indicators of their stability/degradability in the biological environment. This approach proved to be valuable and the literature offers numerous examples of nanomedical studies based on the combination of TEM with various analytical methods. The biodistribution of polylactide nanoparticles loaded with copper chlorophyll as a novel intrinsic contrasting agent were investigated in vivo [137] by TEM and energy-dispersive X-ray (EDX) spectroscopy, verifying the presence of metal ions inside the polymer structure in the tissues. TEM combined with EDX spectroscopy was applied also to investigate the intracellular distribution and chemical composition of silver, iron and TiO_2_ nanoparticles [138,139,140,141,142]. STEM and energy-filtered transmission electron microscopy (EFTEM, which allows image contrast enhancement as well as a chemical/elemental quantification and mapping of the sample) were used to analyze the 3D distribution of carbon-based nanoparticles within whole cells [143]. Advanced TEM methods (conventional, EFTEM and EM tomography) were used in combination with light microscopy to study the distribution of different nanoparticles in tissues and cells [144]. STEM, electron tomography and EDX chemical analyses were performed on cryosections of HeLa cells to investigate iron oxide nanoparticles [145]. An X-ray microanalysis was applied to detect silver nanoparticles in zebrafish organs [146] and STEM, X-ray microanalysis and EDX mapping were applied to study the uptake of metal oxide or gold nanoparticles by macrophages [147,148]. TEM, STEM and EDX spectroscopy were used to detect and analyze the chemical composition of carbon and metal-bearing nanoparticles inhaled and found in the cells of placental tissues [149]. The distribution of TiO_2_ or iron nanoparticles in cells and tissues was described by combining TEM and electron energy loss spectroscopy/electron spectroscopic imaging (ESI/EELS), which provided information on the elemental composition of the sample and the unequivocal detection of the nanoconstructs [150,151]. EELS measurements were also combined with STEM to detect magnetic nanoparticles in cancer cells [152].

## 4. Ultrastructural Histochemistry in Nanomedical Research

**Figure 1:**
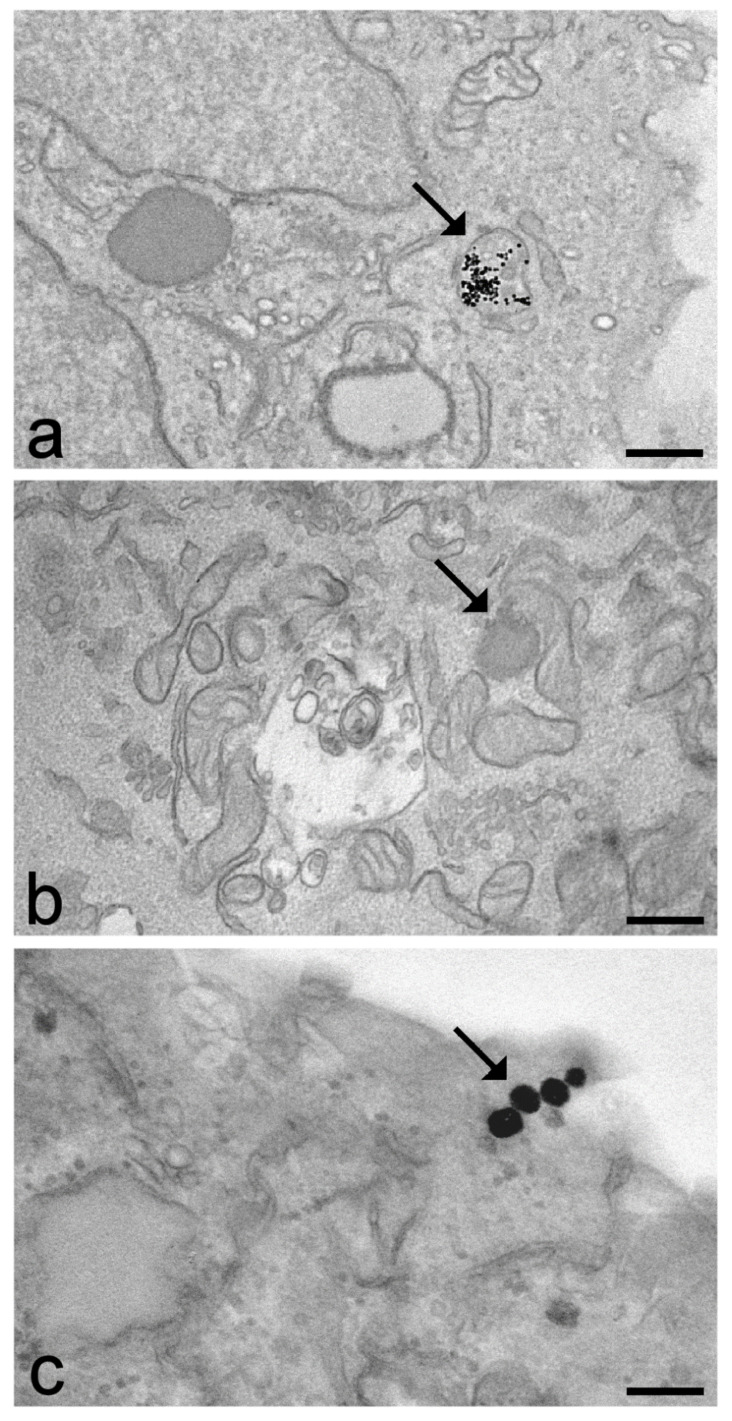
Transmission electron micrographs of cells incubated for 24 h with different types of nanoparticulates (arrows): iron-based nanoparticles in adipose tissue stem cells (**a**), poly(lactic-co-glycolic acid) nanoparticles in C2C12 cells (**b**) and liposomes in HeLa cells (**c**). All cell samples were fixed with aldehydes, post-fixed with osmium tetroxide, embedded in epoxy resin and stained with uranyl acetate to enhance the image contrast. Note the low electron density of the polymeric nanoparticle in (**b**) that makes it hardly detectable in the intracellular milieu. Conversely, the intrinsic electron density of iron (**a**) and the binding of osmium tetroxide to the lipid components of liposomes (**c**) make the particulates in (**a**,**c**) clearly recognizable inside the cell. Bars: 200 nm.

As evident from the above-cited literature, most of the studies performed by TEM concerned inorganic nanoconstructs. TEM nanoparticulates containing heavy atoms (e.g., gold, silver, iron) are strongly electron-dense and are thus easily visible inside cells and tissue in the absence of procedures for contrast enhancement (Figure 1a). On the contrary, it is extremely difficult to detect nanoparticles made of organic materials in the biological environment as their components (e.g., lipids, chitosan, poly(lactic-co-glycolic acid), albumin) contain atoms of a low mass and show a moderate electron density and thus are hardly distinguishable from the cell and tissue components (Figure 1b). 

The visibility in TEM of many lipid-based nanoconstructs may be efficiently improved by the application of osmium tetroxide (Figure 1c) [60,72,78,99,100,153], which induces the formation of an intensely electron-dense reduction product thanks to its addition to the double carbon-to-carbon bonds present in the lipid molecules [154].

The low electron density of polymeric and proteinaceous nanoparticles may be increased by applying specific histochemical procedures. 

**Figure 2:**
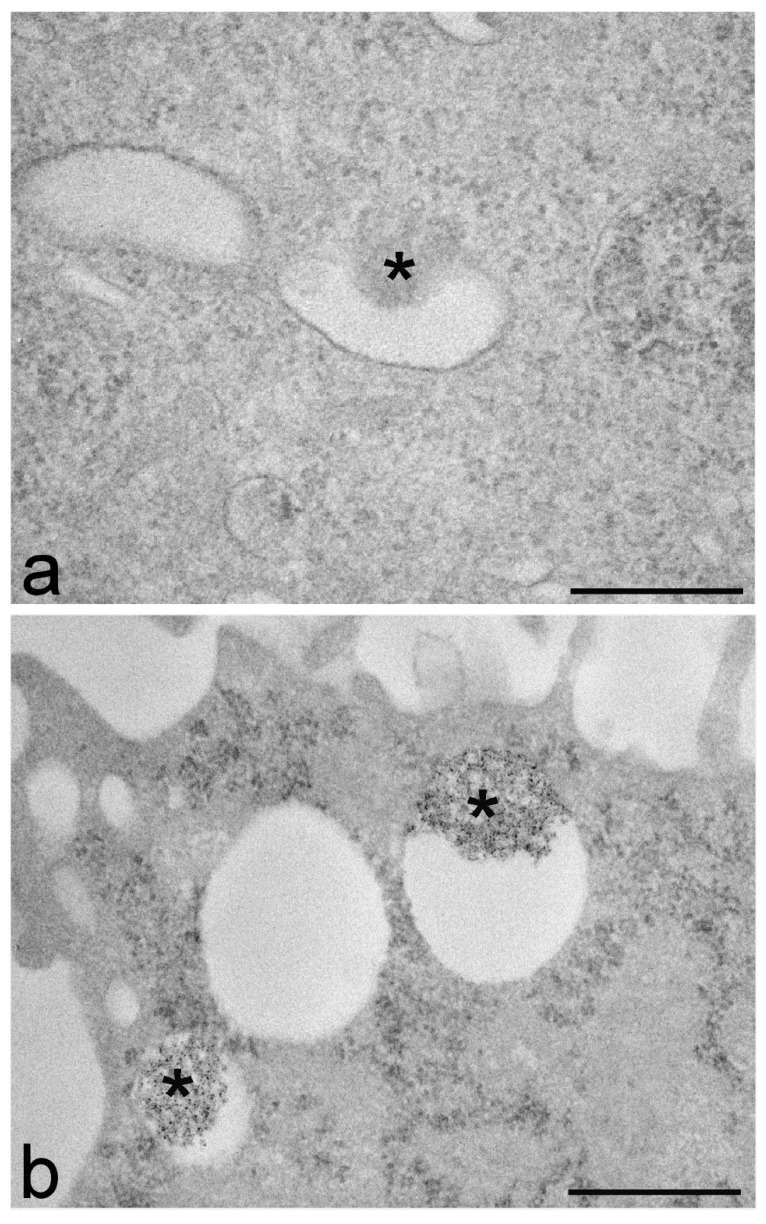
Transmission electron micrographs of B50 cells incubated for 24 h with chitosan-based nanoparticles (asterisks). The sample in (**a**) was fixed with aldehydes, post-fixed with osmium tetroxide and embedded in epoxy resin and the sample in (**b**) was fixed with aldehydes, submitted to DAB photooxidation, post-fixed with osmium tetroxide and embedded in epoxy resin. Both samples were stained with uranyl acetate. In (**a**), the nanoparticle inside the endosome is hardly recognizable due to its weak contrast whereas in (**b**), the electron-dense reaction product of DAB photooxidation makes the nanoparticles clearly visible. Bars: 500 nm. Image in (**b**) from Malatesta et al. [161].

Nanoparticles are usually labelled with various fluorophores to make them visible in fluorescence microscopy. By applying a histochemical technique for the photooxidation of diaminobenzidine (DAB) [155], a fluorescent signal may be converted into a stable reaction product detectable in bright-field microscopy as a brownish precipitate as well as in TEM as an electron-dense finely granular deposit. DAB photooxidation has been widely used in the past for ultrastructural studies on neuronal networks [156,157,158] and intracellular organelle dynamics [159,160] and has proved to be a suitable method also for the ultrastructural detection of fluorescently labelled nanoparticles [161] (Figure 2). 

This technique also allowed the detection of nanoparticle remnants inside secondary lysosomes and residual bodies after they became morphologically unrecognizable due to an enzymatic action [87,161,162].

**Figure 3:**
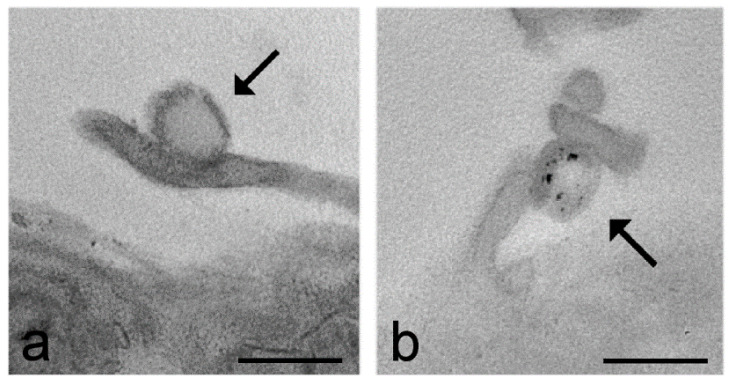
Transmission electron micrographs of C2C12 cells incubated for 2 h with hyaluronic acid-based nanoparticles (arrows). The sample in (**a**) was fixed with aldehydes, post-fixed with osmium tetroxide and embedded in epoxy resin and the sample in (**b**) was fixed with aldehydes, submitted to Alcian blue staining, post-fixed with osmium tetroxide and embedded in epoxy resin. Note the low electron density of the nanoparticles in the conventionally processed sample (**a**) and their increased visibility after the Alcian blue staining (**b**). Bars: 200 nm. Images from Carton et al. [90].

DAB photooxidation is, however, inappropriate when the fluorescently labelled nanoparticles are to be detected inside cells or tissues with a high autofluorescence. Alternative histochemical methods are, therefore, necessary to unequivocally reveal the nanoconstructs at an ultrastructural level. In a recent work, hyaluronic acid-based nanoparticles [163] were made recognizable in TEM by adapting the long-established Alcian blue staining [91] (Figure 3) that was originally proposed to reveal glycosaminoglycans in tissue sections [164].

Nanoparticles may also be identified by TEM by means of immunoelectron microscopy, which allows the detection of specific molecules through their binding with colloidal gold-conjugated antibodies [165]. Polyplexes containing digoxigenin-labelled DNA were localized intracellularly by TEM by using immunogold labelling [166]. Drug-loaded chitosan nanoparticles were immunolabelled by both fluorescence microscopy and TEM by an antibody recognizing the loaded drug; this procedure was suitable not only for detecting the nanoconstructs inside the cells but also for following the intracellular relocation of the released drug [167]. Immunoelectron microscopy was applied also to recognize cell-derived nanovesicles by labelling their membrane markers [168,169,170] or to investigate the association of nanoparticles with specific cellular proteins [144].

It is worth noting that DAB photooxidation and post-embedding immunoelectron microscopy may successfully be combined [171], thus opening the possibility of detecting several antigens on a single section of a photooxidized sample.

## 5. Conclusions

Research in nanomedicine has achieved great progress thanks to the application of imaging techniques often combined with multimodal approaches. Each technique has advantages and limitations and can provide only a piece of information. In this context, TEM is irreplaceable for studying the interaction of nanoconstructs with the tissue and cell components thanks to its high resolution. However, its successful role in nanomedical research is also due to the ingenious and tenacious work of electron microscopists to develop experimental methods and technological tools to overcome the intrinsic limitations of TEM. For instance, when nanoparticles are to be detected inside organs ex vivo, an adequate sampling can be obtained by taking several small tissue portions suitable for a TEM analysis from different organ parts. Taking samples at different times after nanoparticle administration allows dynamic evidence to be obtained of their uptake, relocation and clearance whereas using the tomographic approach, it is possible to obtain reliable 3D reconstructions of their location inside tissues and cells. The simultaneous application of correlative light and electron microscopy or at least of complementary light and electron microscopy techniques is always helpful to exhaustively describe the fate of nanoconstructs in the biological milieu. When the nanoconstructs are made of chemical constituents that are not usually present in the biological material, their occurrence (as well as the presence of their remnants) may be detected using microanalytical techniques such as EDX spectroscopy, ESI or EELS. When nanoparticles are made of organic components that make their ultrastructural detection challenging by both morphological and analytical approaches, traditional histochemical techniques may be applied whereas histochemical or immunohistochemical methods make it possible to visualize the nanoconstructs, their carried drugs and the molecular constituents of the subcellular structures they interact with. 

No doubt, in the years to come, there will be room for a wider application of the ultrastructural techniques in nanomedicine; the multiple endocytic processes involved in nanoparticulate internalization have been only partially explained, especially in relation to the role of membrane receptors. The mechanisms responsible for the uptake of lipid-based nanoparticles are still poorly known and there is a lack of convincing structural evidence. The phenomenon of the endosomal escape, of chief importance for drug delivery, and its consequences on cell viability need to be elucidated and the occurrence of several nanoconstructs inside the cell nucleus requires an in-depth investigation to clarify their effects on the fine organization and activity of the nuclear subdomains in DNA duplication and RNA transcription and splicing. TEM techniques promise to play a primary role in these and other studies; not only they will provide conclusive morphological evidence but they will also help to obtain a finely accurate molecular analysis in situ, enabling the evaluation of the effects of nanoconstruct administration on the structural and functional features of cells and tissues.
